# An Automated Microfluidic System for Haemostasis Assessment in Cirrhosis With Thrombocytopenia

**DOI:** 10.1111/liv.70871

**Published:** 2026-09-14

**Authors:** Niccolò Bitto, Giulia Tosetti, Armando Tripodi, Anna Lecchi, Silvia La Marca, Lidia Padovan, Erica Scalambrino, Marigrazia Clerici, Camilla Caputo, Martina Lucà, Massimo Primignani, Anna Ludovica Fracanzani, Pietro Lampertico, Flora Peyvandi, Vincenzo La Mura

**Affiliations:** ^1^ Foundation I.R.C.C.S. Ca’ Granda, Ospedale Maggiore Policlinico Angelo Bianchi Bonomi Hemophilia and Thrombosis Center Milan Italy; ^2^ Foundation I.R.C.C.S. Ca’ Granda, Ospedale Maggiore Policlinico, Gastroenterology and Hepatology Division Milan Italy; ^3^ Foundation Luigi Villa Milan Italy; ^4^ Department of Pathophysiology and Transplantation University of Milan Milan Italy; ^5^ Foundation I.R.C.C.S. Ca’ Granda, Ospedale Maggiore Policlinico, Unit of Internal Medicine and Metabolic Diseases Milan Italy; ^6^ CRC “A. M. and A. Migliavacca” Center for Liver Disease, Department of Pathophysiology and Transplantation University of Milan Milan Italy

**Keywords:** cirrhosis, coagulopathy, flow chamber, FVIII, platelets

## Abstract

**Background & Aims:**

Conventional laboratory tests do not capture platelet–vessel wall interactions (primary haemostasis) occurring in vivo, limiting guidance before invasive procedures. This is relevant in patients with thrombocytopenia, hallmark of advanced cirrhosis. Microfluidic assays may overcome these limitations. The Total Thrombus Analysis System (T‐TAS) is a flow chamber that can be adapted for thrombocytopenic samples when equipped with the HD‐CHIP. The CirTAS study aimed to assess T‐TAS reproducibility and the ability to identify a disease‐severity gradient in patients with cirrhosis and thrombocytopenia.

**Methods:**

Eighty‐one patients with cirrhosis and thrombocytopenia were enrolled (20 Child–Pugh A, 41 B/C and 20 B/C with bacterial infection). T‐TAS measurements were performed using the HD‐CHIP. Reproducibility was assessed by intraclass correlation coefficients. Thromboelastometry (ROTEM) parameters were analysed in parallel. Multivariable and piecewise regression analyses were performed to test factors influencing T‐TAS parameters.

**Results:**

T‐TAS showed high reproducibility, with intraclass correlation coefficients ≈0.90 for all parameters (*p* < 0.001), even in platelet counts < 50 × 10^9^/L. Thrombus formation under flow declined with worsening clinical status and presence of bacterial infection (*p* < 0.05). Alongside, ROTEM showed a trend towards hypocoagulability with increasing disease severity and correlated with T‐TAS parameters. Platelet count and factor VIII (FVIII) were independent determinants of the most representative T‐TAS parameters. Exploratory analyses detected a trend towards a stronger platelet count‐T‐TAS association at 30–50 × 10^9^/L.

**Conclusions:**

T‐TAS is reproducible in thrombocytopenic cirrhosis and is associated with reduced thrombus formation alongside disease progression and bacterial infection. Platelet count and FVIII are the main determinants of thrombus formation assessed by T‐TAS.

## Key Points

- T‐TAS with the HD‐CHIP showed high reproducibility in patients with cirrhosis and thrombocytopenia, including those with platelet counts below 50 × 10^9^/L.
- Thrombus formation under flow declined with worsening disease severity and bacterial infection; ROTEM showed a parallel trend towards hypocoagulability and correlated with T‐TAS parameters.
- Platelet count and factor VIII were independent determinants of T‐TAS parameters.
- T‐TAS is a candidate laboratory tool for assessing primary haemostasis in cirrhosis and warrants prospective validation against hard clinical endpoints.

## Lay Summary

Low platelet count is common in cirrhosis, but current laboratory tests do not fully reflect how blood clotting works in these patients, making it difficult to predict bleeding risk before procedures. In this study, we show that a new automated microfluidic test provides reproducible measurements even in patients with advanced cirrhosis and severe thrombocytopenia. This approach, if validated, may help improve the evaluation of bleeding risk in clinical practice.

**Abbreviations**
ACLF: acute‐on‐chronic liver failure
AKI: acute kidney injury
AUC: area under the pressure–time curve
CFT: clot formation time
CRP: C‐reactive protein
CT: clotting time
FVIII: factor VIII
HCC: hepatocellular carcinoma
ICCa: intraclass correlation coefficient for absolute agreement
ICCc: intraclass correlation coefficient for consistency agreement
LBP: lipopolysaccharide‐binding protein
MCF: maximum clot firmness
OST: occlusion start time
OT: occlusion time
PC: protein C
PT: prothrombin time
ROTEM: Rotational thromboelastometry
sCD40L: soluble CD40 ligand
TEG: thromboelastography
TRALI: transfusion‐related acute lung injury
T‐TAS: Total Thrombus Analysis System
VWF: von Willebrand factor
VWF:Ag: von Willebrand factor antigen
VWF:RCo: von Willebrand factor ristocetin cofactor activity

## 1 Introduction

Haemostasis is a complex and tightly regulated mechanism that operates in vivo through primary haemostasis and coagulation. Primary haemostasis consists of platelet‐vessel wall interaction and platelet aggregation at the site of vascular injury and is mediated by the multimeric adhesive protein von Willebrand factor (VWF). Coagulation consists of thrombin formation and fibrin deposition, mediated by the opposing driving forces of pro‐ and anticoagulants. Cirrhosis is characterized by decreased levels of pro‐ as well as anticoagulants, thrombocytopenia and increased levels of VWF and factor VIII (FVIII). Pathophysiological studies have consistently demonstrated that this peculiar situation leads to a rebalanced haemostasis [1, 2] that probably explains the relatively low risk of bleeding in patients with cirrhosis undergoing surgical/invasive procedures [3]. Furthermore, the fragile and unstable balance of haemostasis explains the paradox of bleeding or thrombosis, which are occasionally recorded in patients depending on different clinical conditions [4]. Complications like bacterial infections, acute kidney injury (AKI), acute decompensation and progression to acute‐on‐chronic liver failure (ACLF) may dynamically perturb the rebalanced haemostatic equilibrium [5, 6, 7]. In particular, bacterial infections have been associated with a persistent impairment in primary haemostasis [6], whereas other conditions, such as AKI, may induce more transient and potentially reversible changes [7]. Accordingly, patients with similar platelet counts may exhibit different haemostatic phenotypes depending on the clinical context [8, 9]. This may explain why thrombocytopenia, despite being the most frequent haemostatic abnormality observed in cirrhosis, remains a controversial predictor of bleeding [10]. Furthermore, laboratory assays that might be used to establish threshold levels of platelet function and numbers, suitable to prevent spontaneous or perioperative bleeding, are not available. Indeed traditional tests for primary haemostasis, such as platelet count and or platelet aggregometry capture only isolated aspects of the balance operating in vivo and are poor predictors of bleeding [11, 12, 13], thus representing a limitation to guide the management of patients with cirrhosis undergoing invasive procedures for diagnostic or therapeutic purposes [12, 13]. The most recent international guidelines discourage testing patients with cirrhosis with traditional haemostasis assays before invasive procedures [14, 15, 16, 17] and are against pre‐emptive therapies targeting platelets or haemostatic factors [18]. This notwithstanding, there is still an entrenched and unjustified practice to correct primary haemostasis, coagulation and/or fibrinolysis before invasive procedures [19]. This may be driven by legal concerns and is generally based on arbitrary cut‐off of platelet count, prothrombin time (PT) and other conventional tests, although it is widely recognized that they do not represent the complex pathophysiology operating in vivo [20]. All these observations explain the mismatch between expert recommendations and the general clinical practice and highlight the importance of developing new laboratory tests to guide clinical decisions in patients with cirrhosis awaiting surgical/invasive procedures [13, 19]. Thromboelastography/rotational thromboelastometry (TEG/ROTEM) are global haemostasis tests performed on whole blood, that may offer a more integrated view of haemostasis than traditional laboratory tests [21]. However, TEG/ROTEM in their current format have some limitations [22]. They are static tests which are unable to mimic in vitro the hemodynamic shear forces that govern the in vivo contribution of VWF to primary haemostasis, by promoting platelet adhesion and aggregation and to the coagulation pathway by concentrating the release of FVIII, which is carried by circulating VWF [23]. These observations make ROTEM/TEG not adequately sensitive to the VWF results and highly dependent on platelet count [24]. Therefore, the ideal laboratory test in cirrhosis should not neglect the measurement of the VWF contribution to thrombus generation. Microfluidic chambers represent an advanced laboratory technology that mimics in vitro the flow conditions operating in vivo, thus representing much better than any other static laboratory procedure the complex interplay between vessel wall, platelets and VWF in primary haemostasis [25]. The Total Thrombus Analysis System (T‐TAS) is an automated, pump‐driven microfluidic device based on this technology. It dynamically monitors in vitro thrombus formation of blood samples by recording pressure changes of a collagen‐ and tissue factor–coated channel [26, 27]. The HD‐CHIP, a dedicated cartridge specifically designed for thrombocytopenic blood (platelet counts 10–90 × 10^9^/L), uses channel dimensions, shear rate and low concentrations of tissue factor to maximize platelet margination, platelet activation and VWF‐mediated adhesion to collagen despite low platelet counts [28]. Although designed to assess primary haemostasis under flow conditions, the presence of tissue factor may also partially reflect activation of the extrinsic coagulation pathway. This approach requires minimal sample handling and may offer standardized measurements [29, 30]. Recently, T‐TAS has been evaluated in 30 patients with cirrhosis (median platelet count 97 × 10^9^/L) and 30 healthy controls using the PL‐CHIP, a collagen‐coated cartridge designed for normal platelet counts [31]. The study demonstrated progressively delayed and impaired thrombus formation with increasing disease severity, despite increasing VWF levels, suggesting a limited compensatory effect of VWF on primary haemostasis in advanced cirrhosis [32]. To date, T‐TAS with HD‐CHIP has been applied in various hematologic and cardiovascular conditions [33, 34], but has never been tested in patients with cirrhosis. Compared with the PL‐CHIP, the specific design of the HD‐CHIP for thrombocytopenic samples potentially enables assessment of thrombus formation across a broader spectrum of cirrhosis severity, including patients with severe thrombocytopenia. The CirTAS study was designed with two objectives: first, to evaluate the reproducibility of T‐TAS with HD‐CHIP in cirrhosis with thrombocytopenia, across a spectrum of clinical severity; second, to assess its ability to capture a disease‐gradient effect on thrombus formation, in comparison with an established point of care global assay such as ROTEM. Additionally, we explored the influence of platelet count, FVIII levels and other laboratory and clinical variables on T‐TAS parameters.

## 2 Methods

### 2.1 Participants and Study Setting

The CirTAS study enrolled, between January 2021 and September 2024, adult patients with a confirmed diagnosis of cirrhosis and a documented history of thrombocytopenia, defined by a platelet count between 10 × 10^9^/L and 90 × 10^9^/L on two separate consecutive measurements before T‐TAS measurement. The study was conducted at the IRCCS Fondazione Ca′ Granda Ospedale Maggiore Policlinico, Milan. Participants were recruited from outpatient clinics, day hospital and inpatient units. The study was approved by the Ethics Committee ‘Milano Area 2’ (protocol code CirTAS 08/2021, approval number 0045354), and all participants provided written informed consent. Patients were excluded if they had received antiplatelet or anticoagulant therapy within the 2 weeks prior to enrolment, had experienced major bleeding (as per International Society on Haemostasis and Thrombosis criteria) in the previous 42 days, had a known extrahepatic malignancy, were on prolonged (3 or more days) antibiotic therapy before testing, or presented with hepatocellular carcinoma (HCC) beyond Milan criteria. Patients were stratified into three clinical groups based on disease severity: Child A, B/C without infection and B/C with bacterial infection. Child A patients were enrolled as outpatients, while B/C were recruited in both outpatient and inpatient settings. This stratification was chosen to capture not only conventional liver disease severity, but also the potential destabilizing effect of acute bacterial infection on the rebalanced hemostatic equilibrium in advanced cirrhosis. Infected patients were all hospitalized and managed per international guidelines. All patients were screened for COVID‐19, and none tested positive at the time of inclusion. Clinical characterization included cirrhosis aetiology, decompensation history and portal hypertension. Acute on chronic liver failure (ACLF) was defined per EASL‐CLIF criteria [35] and decompensation staging was categorized as: no decompensation (absence of previous decompensating events), first decompensation (first episode of ascites, variceal bleeding or hepatic encephalopathy) and further decompensation (occurrence of a second portal hypertension–driven decompensating event after the first decompensation) [36]. Infections were clinically diagnosed, with microbiological data when available [37]. Hemostatic tests were performed within 48 h of antibiotic initiation. Albumin infusion in the 2 weeks preceding the study and portal hypertension prophylaxis were also recorded.

### 2.2 Total Thrombus Analysis System (T‐TAS) and Additional Hemostatic Analyses

The chip features microchannels measuring 0.3 mm width and 50 μm height, coated with pig‐derived type I collagen and rabbit‐derived tissue factor to simulate vascular injury and to optimally activate primary haemostasis. Citrated blood was recalcified with calcium chloride and supplemented with corn trypsin inhibitor before being perfused through the channel at a controlled shear rate of 1200 s^−1^. Thrombus formation was monitored in real time via pressure sensors, and quantified using three parameters: occlusion start time (OST), defined as the time needed to reach a pressure increase of 10 kPa, which evaluates the first phase of primary haemostasis (adhesion); occlusion time (OT), the time to reach 60 kPa (complete channel occlusion), which combines adhesion, thrombus growth and stabilization; and the area under the pressure–time curve (AUC), reflecting the overall thrombotic potential. ROTEM was employed as a comparative method to evaluate the hemostatic profile alongside T‐TAS. ROTEM was performed on citrated whole blood using the ROTEM Delta system with EXTEM or INTEM cartridge, which activates coagulation via the extrinsic or intrinsic pathway, allowing assessment of clot formation. Standard parameters included clotting time (CT), clot formation time (CFT) and maximum clot firmness (MCF) [38]. Additional analyses included markers of in vivo platelet activation (P‐selectin, soluble CD40 ligand (sCD40L)), VWF antigen and activity (VWF:Ag, VWF:RCo), lipopolysaccharide‐binding protein (LBP) as a marker of bacterial translocation and coagulation and anticoagulant factors (FVIII, protein C).

### 2.3 Statistical Analysis

To assess T‐TAS reproducibility and accuracy, at least 20 patients per group were included. There were no data for cirrhosis in the literature that used T‐TAS to estimate the sample size. We calculated the power of the study by simulating an effect of the clinical severity on the time‐to‐occlusion measured by the T‐TAS of 3–6–9 times, with a variance of 25%–50%–100% and a number of 20 or 30 subjects per group. The simulation is reported in Table S1. After an interim analysis of the first 30 consecutive patients included in the study, the observed variability and effect size estimates confirmed the minimum number of 20 patients per group to respect an alpha‐error of 0.05 and a beta‐error of 0.2 for the endpoint. To minimize selection bias for the comparisons between infected and non‐infected Child B/C patients, the recruitment of patients for the Child B/C without infection group continued beyond the target of 20 until the infected group reached the minimum sample size. T‐TAS measurements were performed in duplicate, tested for correlation by the Spearman's coefficient and reproducibility that was assessed by using the intraclass correlation coefficients for absolute and consistency agreement (ICCa and ICCc, respectively) and Bland–Altman plots. Group comparisons were evaluated using the Jonckheere‐Terpstra test for continuous variables and the chi‐squared test for categorical variables. Univariate and multivariate linear regression models were used to identify independent factors associated with thrombus formation; variables significantly associated with T‐TAS parameters in univariate analysis were entered into multivariate models. To explore potential threshold effects of platelet count on T‐TAS parameters, we performed piecewise linear regression with knots set at 30 and 50 × 10^9^/L. This approach allows estimation of separate slopes of the platelet–T‐TAS relationship within predefined intervals (< 30, 30–50, > 50 × 10^9^/L platelets). A *p*‐value < 0.05 was considered statistically significant. Statistical analyses were performed by SPSS Statistics (version 29, IBM Corp., Armonk, NY) and StataNow MP‐parallel edition version 19.5 (StataCorp, College Station, TX). Figures were generated with GraphPad Prism (version 9, GraphPad Software, San Diego, CA) and BioRender (BioRender.com).

## 3 Results

### 3.1 Population Characteristics

We enrolled 81 cirrhotic patients with thrombocytopenia: 20 in Child‐Pugh Class A, 41 B/C without infection and 20 B/C with bacterial infection. Clinical and laboratory data at the time of T‐TAS measurement in three groups are in Tables 1 and 2. In the whole cohort there was a male predominance, with similar liver disease aetiology in all groups. Alcohol‐ and virus‐related cirrhosis were the most common etiologies, accounting for 31% and 30% of cases, respectively. Prior decompensation had occurred in 68% of patients, most commonly due to ascites. Half of the Child A patients had a history of at least one event of decompensation, while one‐third of Child B/C + infection patients had two or more decompensating events (*p* = 0.072). A prior hospitalization for infection was recorded in 21% of patients, without significant differences between groups (*p* = 0.440). Endoscopic signs of portal hypertension were present in nearly half of the whole cohort, without group differences in prophylaxis or prior TIPS placement. Four Child B/C patients (5%) had Milan‐in active HCC. Rates of recent alcohol use were comparable among groups (*p* = 0.633). Child B/C and B/C + infection more frequently exhibited acute kidney injury, tense ascites and hypotension (*p* < 0.001), coherently with the disease severity gradient postulated for the groups. Similarly, encephalopathy was more frequent (*p* = 0.035) and severe in infected patients (*p* = 0.022). Across the groups MELD, MELD‐Na, INR, bilirubin and C‐reactive protein (CRP) levels increased with the clinical severity, while sodium, platelet count (*p* = 0.017), haemoglobin and haematocrit declined. Neutrophil/lymphocyte ratio, a marker of systemic inflammation, was also high (*p* = 0.012). Despite similar mean creatinine levels, the proportion of patients with creatinine ≥ 1.5 mg/dL increased in parallel with group severity (*p* = 0.043). Platelet counts < 50 × 10^9^/L were found in 21 patients (4 in Child A, 9 in B/C, 8 in B/C + infection). ACLF Grade 1–2 was present in 30% of infected B/C patients (*p* < 0.001). Among the infected patients, urinary tract infections were those with the highest prevalence, followed by spontaneous bacterial peritonitis (Table S2). Among the 13 patients with bacterial isolation, 6 had positive urine cultures, 2 had positive blood cultures, and 5 had bacteria isolated from other sites (e.g., skin, ascites). Gram‐positive and Gram‐negative bacteria were equally distributed in patients who were positive for microbiological tests; only 3 patients had multidrug‐resistant bacteria. No fungal/viral infections were detected in our cohort.

**TABLE 1:** Clinical characteristics of the study population.

	Total (*n* = 81)	Child A (*n* = 20)	Child BC (*n* = 41)	Child BC + infection (*n* = 20)	*p* linear trend
Age (years)	60 (35–90)	61 (35–82)	60 (37–90)	61 (40–78)	0.841
Sex (% males)	52 (64%)	13 (65%)	27 (66%)	12 (60%)	0.743
Aetiology of liver disease					
Viral	26 (30%)	6 (30%)	16 (39%)	4 (20%)	0.700
Alcohol	25 (31%)	8 (40%)	10 (24%)	7 (35%)	
Viral + Alcohol	7 (9%)	3 (15%)	2 (5%)	2 (10%)	
Metabolic	12 (15%)	3 (15%)	5 (12%)	4 (20%)	
Other	11 (15%)	0	8 (20%)	3 (15%)	
History of prior decompensation					
No events of decompensation	26 (32%)	10 (50%)	9 (22%)	7 (35%)	0.072
Single event of decompensation	40 (49%)	9 (45%)	24 (59%)	7 (35%)	
Two or more decompensation events	15 (19%)	1 (5%)	8 (19%)	6 (30%)	
Prior ascites	44 (54%)	6 (30%)	26 (61%)	12 (60%)	0.058
Prior PH‐related bleeding	19 (24%)	6 (30%)	10 (24%)	3 (15%)	0.266
Prior HE	24 (30%)	1 (5%)	17 (42%)	6 (30%)	0.085
Prior HRS	4 (5%)	0	4 (10%)	0	1
Prior SBP	5 (6%)	1 (5%)	4 (10%)	0	0.514
Prior non‐SBP infection with hospitalization	17 (21%)	1 (5%)	13 (32%)	3 (15%)	0.440
Varices					
No varices	16 (20%)	4 (20%)	6 (15%)	6 (32%)	0.440
Small	27 (34%)	6 (30%)	16 (39%)	5 (26%)	
Large or previous ligation	37 (24%)	10 (50%)	19 (46%)	8 (42%)	
PHG	37 (46%)	8 (40%)	20 (49%)	9 (47%)	0.641
Gastric varices	6 (8%)	1 (5%)	5 (12%)	0	0.576
GAVE	8 (10%)	3 (15%)	4 (10%)	1 (5%)	0.292
PH‐bleeding prophylaxis					
NSBB	26 (32%)	6 (30%)	12 (29%)	8 (40%)	0.739
EVL	6 (7%)	0	4 (10%)	2 (10%)	
NSBB + EVL	23 (28%)	7 (35%)	12 (29%)	4 (20%)	
TIPS	8 (10%)	1 (5%)	6 (15%)	1 (5%)	0.389
MELD score	16 (7–33)	10 (7–14)	16 (9–33)	21 (11–33)	< 0.001
MELD‐Na score	16 (7–34)	11 (7–16)	17 (9–29)	22 (11–34)	< 0.001
Active HCC within Milan's criteria	4 (5%)	0	2 (5%)	2 (10%)	0.147
Portal vein thrombosis (no/previous/cavernoma)	71/5/5 (88/6/6%)	18/1/1 (90/5/5%)	36/3/2 (88/7/5%)	17/1/2 (85/5/10%)	0.549
Recent alcohol abuse (< 3 months)	10 (12%)	2 (10%)	5 (12%)	3 (15%)	0.633
Ascites					
Just detectable at ultrasound	19 (24%)	3 (15%)	12 (29%)	4 (20%)	< 0.001
Tense or needing diuretics for control	26 (33%)	0	15 (37%)	11 (55%)	
Encephalopathy (yes/no)	8 (10%)	0	4 (10%)	4 (20%)	0.035
Grade I/II/III/IV no. pts	3/2/3/0	0/0/0/0	3/1/0/0	0/1/3/0	0.022
Grade I/II/III/IV % pts	37/25/38/0	0/0/0/0	75/25/0/0	0/25/75/0	
Decompensation staging					
No decompensation	18 (22%)	10 (50%)	5 (12%)	3 (15%)	< 0.001
First decompensation	27 (33%)	9 (45%)	13 (32%)	5 (25%)	
Further decompensation	36 (44%)	1 (5%)	23 (56%)	12 (60%)	
Acute Kidney Injury	12 (15%)	0	5 (12%)	7 (35%)	< 0.001
Mean arterial pressure	85 (62–130)	93 (75–130)	85 (62–107)	80 (72–100)	< 0.001
Cardiac frequency (bpm)	74 (50–110)	71 (60–87)	75 (51–100)	74 (50–110)	0.550
SpO2 (%)	98 (91–100)	98 (96–100)	98 (93–100)	97 (91–99)	0.025
Recent albumin infusion	37 (46%)	1 (5%)	23 (56%)	13 (65%)	< 0.001
ACLF	7 (9%)	0	1 (2%)	6 (30%)	< 0.001
ACLF Grade					
ACLF Grade 1	3 (4%)	0	0	3 (15%)	0.002
ACLF Grade 2	4 (5%)	0	1	3 (15%)	
ACLF Grade 3	0	0	0	0	

Abbreviations: ACLF, acute‐on‐chronic liver failure; GAVE, gastric antral vascular ectasia; HCC, hepatocellular carcinoma; HE, hepatic encephalopathy; HRS, hepato‐renal syndrome; NSBB, non‐selective beta blocker; PH, portal hypertension; PHG, portal hypertensive gastropathy; SBP, spontaneous bacterial peritonitis; TIPS, transjugular intrahepatic portosystemic shunt.

**TABLE 2:** Baseline laboratory parameters at time of T‐TAS execution across cirrhosis severity groups.

	Total (*n* = 81)	Child A (*n* = 20)	Child BC (*n* = 41)	Child BC + infection (*n* = 20)	*p* J‐T for trend
AST (U/L)	48 (16–200)	34 (27–130)	52 (19–200)	70 (16–161)	0.011
ALT (U/L)	29 (10–156)	31 (18–156)	29 (12–70)	25 (10–68)	0.079
GGT (U/L)	47 (15–836)	104 (18–836)	37 (15–107)	45 (15–170)	0.045
ALP (U/L)	119 (27–518)	122 (51–243)	122 (27–518)	113 (57–384)	0.372
Bilirubin (mg/dl)	2.49 (0.37–28.61)	0.85 (0.44–2.60)	2.98 (0.39–8.20)	3.07 (0.37–28.6)	< 0.001
Albumin (g/dl)	3.6 (2.5–4.8)	4.0 (3.5–4.8)	3.4 (2.8–4.5)	3.2 (2.5–4.3)	< 0.001
Creatinine (mg/dl)	0.85 (0.45–4.68)	0.86 (0.59–1.94)	0.90 (0.51–2.35)	0.82 (0.45–4.68)	0.535
Creatinine ≥ 1.5 mg/dl (% patients)	15 (19%)	1 (5%)	8 (20%)	6 (30%)	0.043
INR	1.53 (1.05–3.86)	1.23 (1.05–1.60)	1.55 (1.12–3.18)	1.64 (1.11–3.86)	< 0.001
Sodium (mEq/l)	138 (125–144)	140 (135–143)	138 (132–143)	137 (125–144)	0.008
CRP (mg/dl)	1.18 (0.01–11.27)	0.09 (0.01–1.29)	0.57 (0.10–5.05)	2.91 (0.33–11.27)	< 0.001
WBC (x10^9^/L)	4.25 (0.9–15.90)	4.35 (1.90–6.60)	3.75 (1.8–9.60)	4.85 (0.90–15.90)	0.362
Haemoglobin g/dl	10.6 (7.2–16.4)	12.5 (9.4–16.4)	10.5 (7.3–16.3)	9.35 (7.2–13.4)	< 0.001
Haematocrit (%)	31.7 (15.8–48.0)	38.1 (28.9–48.0)	30.4 (15.8–47.7)	27.1 (21.3–38.0)	< 0.001
Reticolocytes (%)	2.69 (0.73–9.43)	1.99 (0.80–3.07)	3.06 (1.37–7.30)	2.66 (0.73–9.43)	0.003
Platelets (×10^9^/L)^a^	63 (20–119)	72 (28–119)	62 (26–116)	58 (20–104)	0.017
Neutrophil (%)	62 (25–90)	61 (42–74)	60 (25–81)	70 (47–90)	0.012
Lymphocytes (%)	21 (6–65)	23 (12–45)	21 (9–49)	17 (6–65)	0.012
N/L ratio	2.95 (0.66–15)	2.65 (0.95–6.00)	2.95 (0.66–8.78)	3.88 (1.29–15)	0.012

Abbreviations: CRP, C‐reactive protein; J‐T, jonckheere terpstra test; N/L ratio, neutrophil to lymphocytes ratio; WBC, white blood cell.

^a^ 13 patients had a platelet count at the time of T‐TAS analysis of 104 × 10^9^/L (92–119 × 10^9^/L) despite screening results that indicated a platelet count below 90 × 10^9^/L.

### 3.2 Platelets, Coagulation Factors and von Willebrand Factor

Blood analyses for additional markers of haemostasis and inflammation along with platelet count are in Table 3. Platelet count declined progressively with increasing disease severity (*p* = 0.017). Both antigenic (VWF:Ag) and functional (VWF:RCo) levels, as well as the VWF:RCo/VWF:Ag ratio, increased across groups (*p* = 0.048, *p* < 0.001 and *p* = 0.034, respectively). FVIII levels were comparable among groups, but the FVIII/Protein C (PC) ratio was higher in Child B/C and B/C + infection, consistent with a progressive reduction of PC activity (*p* < 0.001). Fibrinogen levels also decreased with advancing disease (*p* < 0.001). There was a non‐significant trend of elevated LBP levels in infected Child B/C patients, while Child A patients showed intermediate values. Similarly, sCD40L and P‐selectin levels did not differ significantly between groups.

**TABLE 3:** Von Willebrand factor and clotting factors, markers of in vivo platelet activation and inflammation.

	Total (*n* = 81)	Child A (*n* = 20)	Child BC (*n* = 41)	Child BC + infection (*n* = 20)	*p* J‐T for trend
Platelets (×10^9^/L)	63 (20–119)	72 (28–119)	62 (26–116)	58 (20–104)	0.017
VWF:Ag (U/dL)	420 (118–1050)	360 (219–731)	420 (118–988)	478 (242–1050)	0.048
VWF:Rco (U/dL)	332 (84–555)	267 (137–395)	339 (84–512)	385 (211–555)	< 0.001
RCo/Ag	0.74 (0.39–1.56)	0.71 (0.39–0.87)	0.76 (0.52–1.05)	0.78 (0.49–1.56)	0.034
FVIII (act %)	170 (90–402)	163 (127–215)	183 (90–273)	174 (106–402)	0.562
Protein C (act %)	31 (3–92)	56 (23–92)	25 (4–62)	22 (3–59)	< 0.001
FVIII/Protein C	5.3 (1.6–107.7)	3.15 (1.6–6.5)	6.6 (2–37.8)	7.8 (3.3–108)	< 0.001
Fibrinogen (mg/dl)	201 (54–404)	251 (140–404)	172 (54–355)	171 (67–351)	< 0.001
LBP (ng/ml)	18.00 (5.77–79.43)	20.74 (8.47–28.70)	15.63 (5.77–43.21)	24.57 (8.99–79.43)	0.550
SCD40l (ng/ml)	0.86 (0.41–59.95)	0.92 (0.50–18.15)	0.82 (0.46–59.95)	0.89 (0.41–22.05)	0.903
P‐selectin (ng/ml)	81.85 (17.80–470.00)	79 (42–212)	85.1 (17.8–280.7)	69.1 (32.3–470.0)	0.415

Abbreviations: Act, activity; FVIII, Factor VIII; J‐T, jonckheere terpstra test; LBP, lipopolysaccharide binding protein; RCO/Ag, VWF:RCo/VWF:Ag ratio; SCD40l, soluble CD40 ligand; VWF:Ag, von Willebrand Factor Antigen; VWF:Rco, von Willebrand Factor Ristocetin Cofactor.

### 3.3 Reproducibility of T‐TAS HD‐CHIP

T‐TAS parameters measured in duplicate were correlated (*r* = 0.881 for OST, *r* = 0.898 for OT and *r* = 0.891 for AUC; all *p* < 0.001). Median absolute differences between paired measurements were minimal: 0.13 min for OST, 0.18 min for OT and −12 units for AUC. The intraclass correlation coefficient (ICC) for absolute and consistency agreement was ≥ 0.9 for all T‐TAS parameters (*p* < 0.001). Full reproducibility data are in Table 4. Bland–Altman plots (Figure S1) further supported the reliability of the measurements, with a narrow dispersion of differences across the range of values. In addition, when restricting the analysis to patients with platelet counts below 50 × 10^9^/L, median differences were comparable to those observed in the overall cohort, and ICC values consistently exceeded 0.90 for both absolute agreement and consistency across all T‐TAS parameters.

**TABLE 4:** Reproducibility of T‐TAS parameters.

	OST (min)	OT (min)	AUC (units)	*p*
In all patients				
Absolute differences median (min‐max)	0.13 (−3.01–5.88)	0.18 (−4.74–5.20)	−12 (−322–254)	—
ICCa (95% CI)	0.899 (0.846–0.934)	0.907 (0.859–0.939)	0.951 (0.924–0.968)	< 0.001
ICCc (95% CI)	0.901 (0.850–0.935)	0.907 (0.859–0.939)	0.908 (0.860–0.940)	< 0.001
In patients with platelets below 50 × 10^9^/L				
Absolute differences median (min‐max)	0.14 (−3.01–5.04)	0.14 (−4.74–5.19)	−11 (−319–254)	—
ICCa (95% CI)	0.925 (0.825–0.969)	0.921 (0.817–0.967)	0.937 (0.851–0.974)	< 0.001
ICCc (95% CI)	0.959 (0.900–0.984)	0.919 (0.811–0.966)	0.966 (0.916–0.986)	< 0.001

Abbreviations: AUC, area under the curve; CI, confidence interval; ICCa, intraclass correlation coefficient—absolute agreement; ICCc, intraclass correlation coefficient, consistency of agreement; OST, occlusion starting time; OT, occlusion time.

### 3.4 Accuracy in Detecting a Disease‐Gradient Effect Among the Groups

#### 3.4.1 Thrombus Formation Under Flow

**FIGURE 1:**
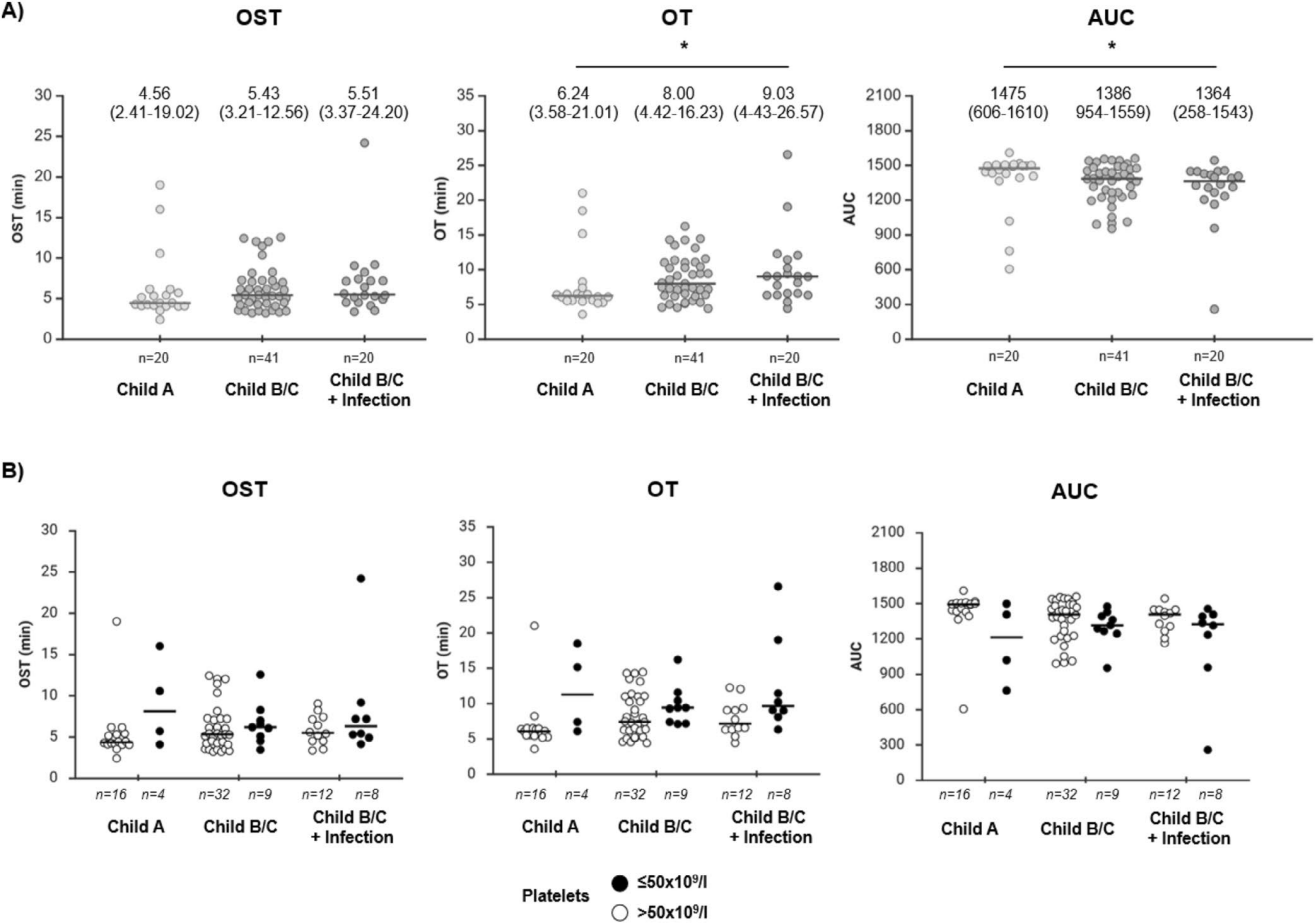
T‐TAS parameters between groups and platelet thresholds. (A) Occlusion time (OT) and Area under the Curve (AUC) show significantly slower and reduced whole blood clot formation under flow alongside disease severity, whilst occlusion starting time (OST) shows a non‐significant trend. (B) T‐TAS parameters, compared by clinical group and platelet threshold of 50 × 10^9^/L show no significant differences. *p < 0.05.

T‐TAS parameters reflected a progressive impairment of thrombus formation with increasing disease severity. Specifically, occlusion time (OT) was significantly prolonged (*p* = 0.032) and area under the curve (AUC) significantly reduced (*p* = 0.025) across severity groups (Figure 1, panel A), while occlusion start time (OST) showed a non‐significant trend (*p* = 0.128). In the subgroup analysis stratified by a platelet count threshold of 50 × 10^9^/L, no statistically significant differences emerged.

#### 3.4.2 Rotational Thromboelastometry (ROTEM)

ROTEM results are in Table S3 (panel A, B, C). Data generally indicated a tendency towards slower clot formation in Child B/C patients, regardless of infection status. However, this tendency was not consistently observed across all parameters, which, in most cases, did not reach statistical significance. For CT and CFT, which represent the time to clot initiation and its early progression, only EXTEM‐CT showed a non‐significant prolongation between groups (*p* = 0.162). The main ROTEM parameters used in clinical practice (CT, CFT, MCF) correlated with the T‐TAS parameters (*p* < 0.05). (Table S4).

### 3.5 Factors Influencing Thrombus Formation Under Flow: Uni‐ and Multivariate Analysis

Univariate analysis showed that platelet count and FVIII activity were the only variables significantly associated with T‐TAS parameters (Table 5). A higher platelet count was associated with shorter OST (B = −0.038, *p* = 0.022) and OT (B = −0.072, *p* < 0.001), as well as increased AUC (B = 3.242, *p* = 0.002). Similarly, higher FVIII levels were associated with shorter OST (B = −0.016, *p* = 0.031), shorter OT (B = −0.022, *p* = 0.010) and higher AUC (B = 1.086, *p* = 0.023). In multivariate analysis adjusting for the group of disease severity, both platelet count and FVIII remained independently associated with thrombus formation (Table 6). Platelet count was significantly associated with OT (B = −0.057, *p* = 0.003) and AUC (B = 2.553, *p* = 0.018), while FVIII retained an independent association with OT (B = −0.017, *p* = 0.041). This finding was also consistent in a multivariable analysis that included platelet count, FVIII and MELD score (data not shown).

**TABLE 5:** Univariate analysis of factors influencing T‐TAS parameters.

	OST				OT				AUC			
	B	95% CI		*p*	B	95% CI		*p*	B	95% CI		*p*
Platelets (×10^9^/L)	−0.038	−0.070	−0.006	0.022	−0.072	−0.106	−0.037	< 0.001	3.242	1.275	5.208	0.002
VWF:Ag (U/dL)	−0.004	−0.008	0.000	0.062	−0.003	−0.008	0.001	0.168	0.205	−0.052	0.462	0.116
VWF:RCo (U/dL)	−0.007	−0.015	0.001	0.084	−0.006	−0.015	0.003	0.218	0.357	−0.132	0.847	0.150
RCO/Ag	1.058	−4.277	6.393	0.694	0.758	−5.377	6.894	0.806	−57.069	−391.932	277.794	0.735
sCD40L (ng/ml)	0.046	−0.052	0.145	0.351	0.068	−0.044	0.180	0.232	−3.558	−9.707	2.591	0.253
P‐selectin (ng/ml)	0.000	−0.013	0.012	0.961	−0.001	−0.015	0.013	0.867	0.021	−0.749	0.790	0.958
FVIII (act %)	−0.016	−0.031	−0.002	0.031	−0.022	−0.039	−0.005	0.010	1.086	0.153	2.019	0.023
Protein C (act %)	−0.007	−0.048	0.035	0.747	−0.032	−0.079	0.015	0.182	1.205	−1.383	3.793	0.357
FVIII/Protein C	−0.017	−0.078	0.043	0.576	−0.021	−0.091	0.048	0.542	0.882	−2.924	4.688	0.646
Fibrinogen (mg/dl)	−0.001	−0.012	0.009	0.788	−0.009	−0.021	0.002	0.119	0.332	−0.311	0.974	0.308
Albumin (g/dl)	−0.103	−1.644	1.438	0.895	−0.885	−2.645	0.876	0.320	27.751	−68.769	124.272	0.569
Haemoglobin (g/dl)	−0.093	−0.459	0.272	0.613	−0.214	−0.632	0.205	0.313	9.864	−13.023	32.752	0.394
Haematocrit (%)	−0.017	−0.140	0.105	0.776	−0.052	−0.192	0.088	0.460	2.364	−5.283	10.011	0.540
CRP (mg/dl)	−0.069	−0.507	0.370	0.754	−0.100	−0.614	0.414	0.697	2.083	−25.632	29.799	0.881
MELD	0.002	−0.128	0.133	0.973	0.060	−0.089	0.210	0.426	−2.574	−10.753	5.605	0.533
MELD‐Na	−0.019	−0.139	0.101	0.750	0.027	−0.110	0.164	0.697	−0.924	−8.433	6.584	0.807
Decompensation staging	−0.519	−1.520	0.482	0.305	−0.250	−1.407	0.908	0.669	19.682	−43.420	82.785	0.536
ACLF	1.302	−1.502	4.105	0.358	1.035	−2.196	4.267	0.526	−96.135	−271.694	79.424	0.279

Abbreviations: ACLF, acute on chronic liver failure; Act, activity; AUC, area under the curve; CRP, c‐reactive protein; FVIII, Factor VIII; J‐T, jonckheere terpstra test; OST, occlusion starting time; OT, occlusion time; RCO/Ag, VWF:RCo/VWF:Ag ratio; SCD40l, soluble CD40 ligand; VWF:Ag, von Willebrand Factor Antigen; VWF:Rco, von Willebrand Factor Ristocetin Cofactor.

**TABLE 6:** Multivariate analysis of factors influencing T‐TAS parameters.

	OST				OT				AUC			
	B	95% CI		*p*	B	95% CI		*p*	B	95% CI		*p*
Platelets (×10^9^/L)	−0.029	−0.063	0.006	0.103	−0.057	−0.094	−0.020	0.003	2.553	0.454	4.651	0.018
FVIII (%)	−0.014	−0.030	0.002	0.081	−0.017	−0.034	−0.001	0.041	0.871	−0.079	1.821	0.072
Group	0.296	−0.850	1.442	0.608	0.700	−0.526	1.925	0.259	−32.258	−101.829	37.312	0.359

Abbreviations: Act, activity; AUC, area under the curve; CRP, c‐reactive protein; FVIII, Factor VIII; OST, occlusion starting time; OT, occlusion time.

### 3.6 Threshold Effects of Platelet Count on T‐TAS Parameters

**FIGURE 2:**
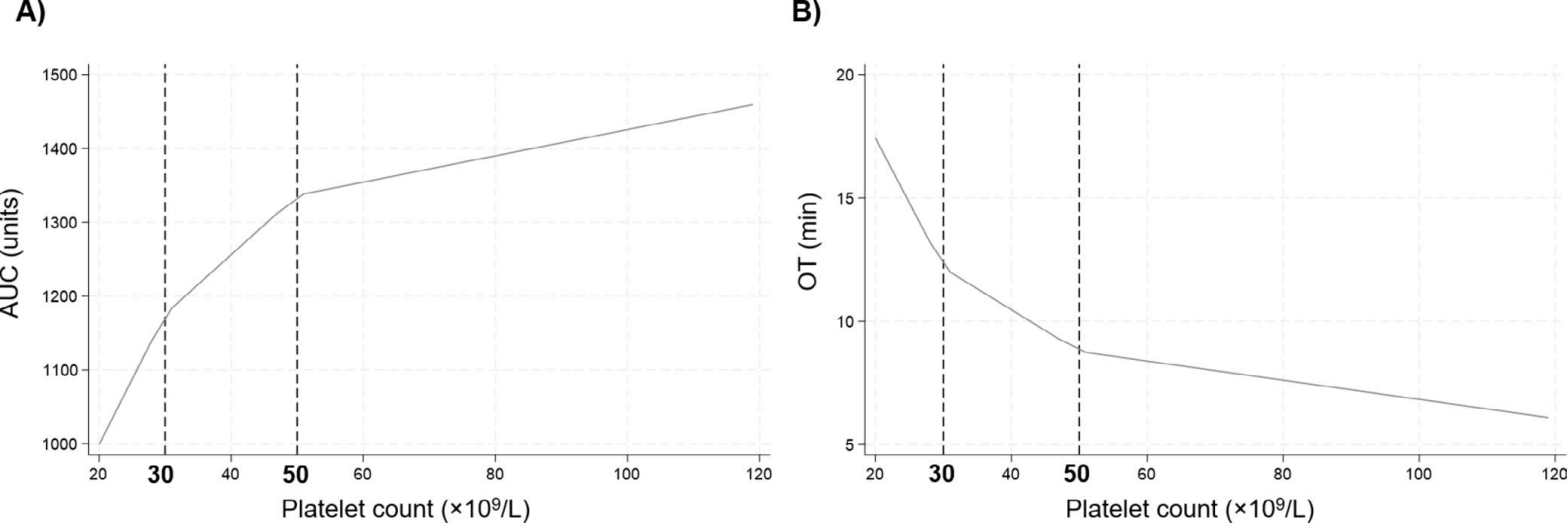
Platelet‐count thresholds and thrombus formation. (A) Relation between platelet count (× 10^9^/L) and area under the curve (AUC); piecewise regression with knots at 30 and 50 × 10^9^/L shows a marginal increase above 50 × 10^9^/L. (B) Relation between platelet count (× 10^9^/L) and occlusion time (OT) with the same knots, showing steeper OT decrease in the 30–50 × 10^9^/L range than plateau > 50 × 10^9^/L. Vertical dashed lines indicate the 30 and 50 × 10^9^/L thresholds.

Exploratory piecewise regression analyses showed a possible non‐linear association between platelet count and T‐TAS parameters across different platelet count ranges, the strongest at low platelet counts. (Figure 2). Occlusion time (OT) showed a trend towards shorter occlusion times with increasing platelet count between 30 and 50 × 10^9^/L (B = −0.17, 95% CI –0.35 to 0.01, *p* = 0.064) with a similar negative trend above 50 × 10^9^/L (B = −0.039, 95% CI –0.078 to 0.00, *p* = 0.051). A marginal increase of AUC was observed at higher platelet counts, although this association did not reach statistical significance (*p* = 0.107), consistent with a plateau of thrombus formation capacity at higher platelet counts. OST did not show significant associations across platelet strata. Full regression coefficients are in Table S5.

## 4 Discussion

In this study, we evaluated the reproducibility and reliability of the T‐TAS device equipped with HD‐CHIP cartridges for testing primary haemostasis in patients with cirrhosis and thrombocytopenia. Results show that the T‐TAS displays excellent reproducibility even at platelet count below 50 × 10^9^/L and identifies impairment in thrombus formation across patients with different disease severity, identifying platelet count and FVIII levels as the main independent determinants of T‐TAS parameters.

Assessing primary haemostasis is complex, as conventional laboratory tests fail to capture the interplay between platelets, VWF and the injured vessel wall [13, 20, 39]. The complexity is more evident in thrombocytopenic cirrhotic patients. Up to now, platelet aggregation, global assays, such as TEG/ROTEM, and thrombin generation in platelet‐rich plasma have contributed substantially to understanding the mechanisms that regulate primary haemostasis [40, 41], but each of the above tests has limitations that prevent application in bleeding‐risk stratification [17, 22, 24]. More recently, other tests have been proposed to aim to overcome the confounding effect of thrombocytopenia. They have shown an association with clinically relevant outcomes in decompensated cirrhosis. In particular, thrombocytopenia‐corrected platelet aggregation (PLT‐ratio) [42] and whole‐blood thrombin generation [43] were associated with further decompensation and procedure‐related bleeding, respectively supporting the potential value of such functional assays beyond platelet count alone. However, all of the above assays are performed under static conditions, which makes it impossible to account for the shear‐stress mediated contribution of VWF to haemostasis. Moreover, many are affected by significant pre‐analytical variability and often require specific corrections for thrombocytopenia [13, 24, 44]. The T‐TAS with HD‐CHIP overcomes several of these limitations [27, 28, 33]. Three important features make the performance of HD‐CHIP superior to conventional tests for primary haemostasis. First, the test cartridge contains exogenous tissue factor, which accelerates thrombin generation on activated platelets. This should compensate for the low platelet count observed in cirrhosis. Second, HD‐CHIP operates under high‐shear flow, which mimics in vivo conditions and optimally activates VWF in vitro. Third, HD‐CHIP requires no handling of blood samples or reagent modifications when platelet counts are in the range of 10–90 × 10^9^/L [28].

This study showed that T‐TAS parameters when evaluated with HD‐CHIP, progressively moved from normal to reduced thrombus formation according to the severity of the disease as well as the presence of acute infection. Indeed, patients with Child‐Pugh B/C and acute infection had significantly longer occlusion times (OT) and reduced area under the curve (AUC) compared with Child‐Pugh A and B/C without infection. These findings suggest that the progression of liver disease, when accompanied by systemic inflammation and organ dysfunction, is associated with reduced in vitro activity of primary haemostasis [4, 45]. In our study, T‐TAS and ROTEM parameters showed a good correlation and were both influenced by disease severity, supporting their potential applicability to assess haemostasis in cirrhosis. However, the high levels of VWF observed in Child‐Pugh B/C patients with infection suggest that dynamic, rather than static, assays may more accurately reflect haemostasis in decompensated cirrhosis. Multivariate analysis confirmed that platelet count and FVIII levels were the main independent determinants of OT and AUC under in vitro conditions of high‐shear. These results are consistent with their roles in platelet adhesion/aggregation and thrombin production on activated platelets, respectively and suggest that the association of reduced thrombus formation with disease severity is largely driven by these underlying biological determinants [46]. Conversely, plasma VWF levels and in vivo platelet activation markers (P‐selectin, sCD40L) were not associated with T‐TAS parameters, likely reflecting the uniformly high VWF concentrations and the consequent platelet activation in the patients included in this study. Indeed, these patients were highly selected because of the inclusion criterion of low platelet count that typically occurs in most advanced stages of cirrhosis. Furthermore, the high shear used by the HD‐CHIP maximizes platelet‐VWF interaction regardless of the plasma levels of VWF [28]. The present findings extend the seminal observations by Lisman et al., which demonstrated that elevated VWF:Ag levels in cirrhosis sustain platelet adhesion under flow, despite reduced functional activity [1]. While that work provided critical mechanistic insight into flow‐dependent primary haemostasis, it relied on a non‐automated experimental system and was not designed for standardized evaluation across clinically predefined patient subgroups. Recently, Abdoul et al. reported progressively impaired thrombus formation using the PL‐chip of the T‐TAS system in patients with cirrhosis, despite markedly elevated VWF antigen and activity levels [31]. Using a collagen‐coated cartridge primarily validated for platelet counts above 100 × 10^9^/L the authors observed reduced thrombus formation across Child‐Pugh stages, suggesting a partial mismatch between circulating VWF levels and effective thrombus formation under flow [32]. Our results extend the observations by Abdoul et al. in a cohort of cirrhotic patients with thrombocytopenia, when evaluated by a cartridge specifically designed for platelet counts between 10 and 90 × 10^9^/L. Taken together, the above findings challenge the hypothesis that elevated circulating VWF may counterbalance the effects of thrombocytopenia on thrombus formation [1]. All in all, the excellent reproducibility of the T‐TAS measurement observed in this study in patients with platelet counts below 50 × 10^9^/L candidates the T‐TAS device with HD‐CHIP as a promising tool for automated, laboratory‐based bleeding‐risk stratification, particularly in thrombocytopenic cirrhotic awaiting invasive procedures [3]. Prospective studies have shown that procedure‐related bleeding in hospitalized patients with cirrhosis, though infrequent (around 8%), is associated with increased 28‐day mortality independent of MELD score [12]. The above considerations emphasize the need for a more accurate identification of patients at risk to reduce unnecessary pre‐emptive interventions and avoid complications such as volume overload that can increase portal pressure [47], transfusion‐related acute lung injury (TRALI) [48] and thrombosis [49, 50]. Currently, major international guidelines discourage routine correction of laboratory abnormalities, including primary haemostasis (i.e., thrombocytopenia/thrombocytopathy) before low‐to‐moderate risk procedures in the absence of robust evidence [14, 17]. A global, reproducible assay such as the T‐TAS could represent a step towards a more rational laboratory‐based decision algorithm, which may (hopefully) contribute to move beyond platelet count alone when assessing procedural bleeding risk in cirrhosis [13, 32].

Despite the above observations confirming the technical advancement of T‐TAS as a tool exploring primary haemostasis in cirrhosis, the study has some limitations. First, the relatively small sample size limited subgroup analyses for clinical variables that could potentially influence haemostasis in cirrhosis. Second, although the HD‐chip demonstrated excellent reproducibility even in patients with severe thrombocytopenia, the number of patients included did not allow adequately powered subgroup analyses based on platelet count. Finally, the study did not correlate T‐TAS parameters with the hard‐clinical end‐point of bleeding, which restricts the immediate translation of our data into clinical practice. However, due to the relatively low rate of bleeding events observed for the most common invasive procedures [12], that goal could be conclusively reached only by large collaborative studies linking T‐TAS parameters to clinically relevant bleeding and decision algorithms for therapies targeting haemostasis. In conclusion, the T‐TAS equipped with HD‐CHIP is a candidate laboratory tool to evaluate primary haemostasis in cirrhosis across disease severity. The test is automated with limited handling of blood samples and reagents. It operates in patients with thrombocytopenia, making it a promising candidate for future investigations aimed at improving laboratory assessment of bleeding risk in cirrhosis.

## Author Contributions

Conceptualization: N.B., V.L.M.; Methodology and statistical analysis: N.B., V.L.M., A.T.; Data collection: N.B. G.T., M.L., C.C; Laboratory analysis: N.B., S.L.M., L.P., E.S., M.C., A.L.; Resources: A.T., F.P.; Writing – original draft: N.B.; Writing – revision for major critical contents: V.L.M., A.L.F., M.P., P.L., A.T., F.P.; Supervision: V.L.M.; Project administration: A.T., F.P.; Funding acquisition: V.L.M., A.T., F.P. All authors approved the final version of the manuscript.

## Funding

The study was (partially) supported by the Italian Ministry of Health—Bando Ricerca Corrente. The Haemostasis & Thrombosis Unit of the Fondazione IRCCS Ca′ Granda Ospedale Maggiore Policlinico is a member of the European Reference Network on Rare Haematological Diseases EuroBloodNet‐Project ID No 101157011. ERN‐EuroBloodNet is partly co‐funded by the European Union within the framework of the Fourth EU Health Programme. Italian Ministry of Education and Research—MUR (‘Dipartimenti di Eccellenza’ Programme 2023–27—Department of Pathophysiology and Transplantation, Università degli Studi di Milano).

## Ethics Statement

The study was approved by the Ethics Committee ‘Milano Area 2’ (protocol code CirTAS 08/2021, approval number 0045354), and all participants provided written informed consent.

## Conflicts of Interest

N.B.: travel grant from Biomarin; G.T.: Symposia/educational meetings: Gilead, Alfasigma; A.T.: no competing interests; A.L.: no competing interests; S.L.M.: no competing interests; L.P.: no competing interests; E.S.: no competing interests; M.C.: no competing interests; C.C.: no competing interests; M.L.: no competing interests; A.L.F.: no competing interests; P.L.: Advisory Board/Speaker Bureau for: Roche Pharma/Diagnostics, Gilead Sciences, GSK, Abbvie, Janssen, Myr, Eiger, Antios, Aligos, Vir, Grifols, Altona, Roboscreen, Bluejay; F.P.: Advisory boards: CSL Behring, Biomarin, Roche, Sanofi, Sobi, Pfizer. Symposia/educational meetings: Takeda, Sanofi, Sobi; M.P.: no competing interests; V.L.M.: Advisory boards: CSL Behring, Biomarin, Pfizer, Alfasigma. Symposia/educational meetings: Biomarin, Gore, Alfasigma. Travel grant: Takeda, Sanofi, Bayer, Roche.

## Supporting information


**Figure S1:** Bland–Altman Plot for T‐TAS parameters.
**Table S1:** Sample size and power: k‐independents samples (ANOVA). Alpha error = 0.05 each simulation.
**Table S2:** Site of infection and microbiological data.
**Table S3:** ROTEM data.
**Table S4:** Correlation between main T‐TAS and ROTEM parameters.
**Table S5:** Piecewise regression coefficients for the association between platelet count and T‐TAS parameters.

## Data Availability

For original data contact the corresponding author, Prof. Vincenzo La Mura (vincenzo.lamura@unimi.it).
