# Supplementary material for: An Automated Microfluidic System for Haemostasis Assessment in Cirrhosis With Thrombocytopenia
Source: Liver Int. 2026 Sep 14;46(10):e70871. doi: 10.1111/liv.70871 (PMC13575452; doi:10.1111/liv.70871)
Supplement: Supplementary file 1 [file LIV-46-0-s001.docx]

**SUPPLEMENTARY MATERIALS**

**FIGURES**

**Supplementary Figure 1 –** Bland-Altman Plot for T-TAS parameters

**TABLES**

**Supplementary Table 1 -** Sample size and power: k-independents samples (ANOVA). Alpha error=0.05 each simulation

| *Effect* | *Variance* | **Power**  N=20 | **Power**  N=30 |
| --- | --- | --- | --- |
| 3 | 25 | 31.0% | 47.2% |
| 3 | 50 | 14.6% | 22.6% |
| 3 | 100 | 7.4% | 10.9% |
| 6 | 25 | 91.6% | 98.8% |
| 6 | 50 | 61.4% | 81.4% |
| 6 | 100 | 31.0% | 47.2% |
| 9 | 25 | 100% | 100% |
| 9 | 50 | 94.9% | 99.4% |
| 9 | 100 | 67.4% | 86.3% |

**Supplementary table 2 –** Site of infection and microbiological data

| **Site of infection** |  |
| --- | --- |
| *Pneumonia* | 3 (15%) |
| *Urinary tract infection* | 6 (30%) |
| *Skin-abscess* | 2 (10%) |
| *Gastrointestinal* | 2 (10%) |
| *Spontaneous bacterial peritonitis* | 5 (25%) |
| *Uncomplicated bacteremia* | 2 (10%) |
| **Patients with microbiological isolation** | 13 |
| **Blood culture positive/performed** | 2/16 |
| **Urine culture positive/performed** | 6/18 |
| **Other cultures** | 5 |
| **Bacterial isolation Gram+/Gram- /Anaerobic** | 6/6/1 |
| **MDR bacteria/total isolates** | 3/13 |

**Supplementary table 3 –** ROTEM data

A) **EXTEM**

|  | **Total**  (n=81) | **Child A**  (n=20) | **Child BC**  (n=41) | **Child BC + Infection**  (n=20) | **P**  *J-T for trend* |
| --- | --- | --- | --- | --- | --- |
| **CT (s)** | 74 (25-119) | 70 (53-89) | 72 (25-119) | 81 (55-119) | 0.162 |
| **CFT (s)** | 167 (47-720) | 142 (85-401) | 189 (47-625) | 167 (93-720) | 0.171 |
| **MCF (mm)** | 49 (27-63) | 53 (35-63) | 45 (28-60) | 49 (27-63) | 0.073 |
| **Abbreviations** – CT, clotting time (s). CFT, clotting formation time (s), MCF, maximum clot firmness (mm). | | | | |  |
|  | | | | |  |

B) **INTEM**

|  | **Total**  (n=81) | **Child A**  (n=20) | **Child BC**  (n=41) | **Child BC + Infection**  (n=20) | **P**  *J-T for trend* |
| --- | --- | --- | --- | --- | --- |
| **CT (s)** | 229 (121-321) | 222 (121-278) | 235 (164-321) | 211 (177-294) | 0.377 |
| **CFT (s)** | 155 (73-1136) | 133 (73-434) | 174 (88-856) | 150 (89-1136) | 0.348 |
| **MCF (mm)** | 49 (24-63) | 51 (34-63) | 44 (25-58) | 49 (24-61) | 0.345 |
| **Abbreviations** – CT, clotting time (s). CFT, clotting formation time (s), MCF, maximum clot firmness (mm). | | | | | |
|  | | | | | |

**Supplementary table 4 –** Correlation between main T-TAS and ROTEM parameters

|  | **OST (min)** | **OT (min)** | **AUC (units)** |
| --- | --- | --- | --- |
| **CT (s)_extem_** | R=0.309  p=0.005 | R=0.412  P<0.001 | R=-0.393  p<0.001 |
| **CFT (s)_extem_** | R=0.213  p=0.058 | R=0.354  P=0.001 | R=-0.300  p=0.007 |
| **MCF (mm)_extem_** | R=-209  p=0.063 | R=-0.364  P<0.001 | R=0.304  p=0.006 |
| **CT (s)_intem_** | R=0.149  p=0.186 | R=0.195  p=0.083 | R=-0.175  p=0.120 |
| **CFT (s)_intem_** | R=0.251  p=0.025 | R=0.312  P<0.001 | R=-0.340  p=0.002 |
| **MCF (mm)_intem_** | R=-0.209  p=0.063 | R=-0.363  P<0.001 | R=0.293  p=0.008 |
| **Abbreviations** – OT: occlusion time. OST: occlusion starting time. AUC: area under the curve. CT, clotting time (s). CFT, clotting formation time (s), MCF, maximum clot firmness (mm). | | | |

**Supplementary table 5 –** Piecewise regression coefficients for the association between platelet count and T-TAS parameters

| **Variable** | **Platelets range (10^9^/L)** | **B** | **95% CI** | **p** |
| --- | --- | --- | --- | --- |
| **OT** | 0–30 | -0.53 | -1.11 ; 0.06 | 0.075 |
| **OT** | 30–50 | -0.17 | -0.35 ; -0.01 | 0.064 |
| **OT** | >50 | -0.039 | -0.078 ; ~0.00 | 0.051 |
| **AUC** | 0–30 | +17.7 | -11.3 ; 46.6 | 0.228 |
| **AUC** | 30–50 | +8.05 | -2.7 ; 18.8 | 0.140 |
| **AUC** | >50 | +1.78 | -0.40 ; 3.95 | 0.107 |
| **OST** | 0–30 | -0.07 | -0.53 ; 0.39 | 0.761 |
| **OST** | 30–50 | -0.11 | -0.30 ; 0.07 | 0.222 |
| **OST** | >50 | -0.019 | -0.054 ; 0.016 | 0.288 |
